# Influence of Work on Andropause and Menopause: A Systematic Review

**DOI:** 10.3390/ijerph181910074

**Published:** 2021-09-25

**Authors:** Margherita Martelli, Laura Zingaretti, Gianmaria Salvio, Massimo Bracci, Lory Santarelli

**Affiliations:** 1 Occupational Health, Department of Clinical and Molecular Sciences, Polytechnic University of Marche, 60126 Ancona, Italy; margherita.martelli@ospedaliriuniti.marche.it (M.M.); l.santarelli@staff.univpm.it (L.S.); 2 Occupational Medicine Unit, Department of Medical and Surgical Specialties, United Hospitals Ancona, 60126 Ancona, Italy; laura.zingaretti@ospedaliriuniti.marche.it; 3 Endocrinology Clinic, Department of Clinical and Molecular Sciences, Polytechnic University of Marche, 60126 Ancona, Italy; g.salvio@pm.univpm.it

**Keywords:** testosterone, estradiol, workplace, workers, job, late-onset hypogonadism

## Abstract

Aging is associated with gender-specific hormonal changes that progressively lead to gonadal insufficiency, a condition which characterizes a minority of men and all women. Work-related factors, such as stress and pollutant exposure, affect gonadal function and can interfere with reproduction in both genders. A systematic review of the PubMed, SCOPUS and EMBASE databases was conducted, according to the Preferred Reporting Items for Systemic Reviews and Meta-Analyses (PRISMA) statement to investigate the effect of occupational factors on andropause and menopause. A total of 26 studies met the inclusion and exclusion criteria: 9 studies evaluated the effects of work on andropause symptoms, 8 studies examined its effects on age at menopause onset, and 9 studies addressed its effects on menopausal symptoms. Work-related factors, such as psychological stress, physical effort, and sleep disorders, showed a significant correlation with andropause manifestations, whereas age at menopause and severity of menopausal symptoms were both influenced by factors such as pesticide exposure, high job strain, and repetitive work. Since work accompanies men and women for most of their lives, it is essential to identify and prevent the risk factors that may affect reproductive health.

## 1. Introduction

Aging is associated with modifications of the hypothalamic–pituitary–gonadal (HPG) axis, which in females lead to menopause and in a variable proportion of males lead to a clinical entity called andropause or, according to more recent terminology, late-onset hypogonadism (LOH).

The physiological age of menopause is on average 46 years; the diagnosis is made retrospectively after at least 12 months of amenorrhea [1]. The Stages of Reproductive Aging Workshop + 10 (STRAW + 10) criteria, which are based on menstrual bleeding patterns, were established in 2001 and updated in 2012 [2]. Vasomotor symptoms affect 80% of women, but their pathophysiology is not clearly understood. Notably, aside from reduced circulating estrogen, increased responsiveness of the hypothalamic thermoregulatory zone to changes in body temperature are believed to be affected by central mechanisms mediated by hormonal stimuli (e.g., high levels of luteinizing hormone (LH) from the pituitary gland) or local factors (e.g., hypothalamic neurokinin B) [2].

Unlike aging women, most men do not experience a dramatic decline in gonadal function. In fact, although morning serum testosterone (T) levels decline progressively over time, only 20% of adults aged 65 years or more have T levels below the normal range for young men [1]. In addition, unlike menopausal symptoms, most of the signs and symptoms of LOH are mild and difficult to distinguish from the effect of aging per se. From a pathophysiological point of view, aging is associated with a progressive reduction in testicular T production as an effect of Leydig cell degeneration and of atherosclerosis of testicular arterioles. However, in most men, an increase in LH compensates for the reduced function of the testis and maintains adequate T levels, preventing the appearance of signs of hypogonadism. Additional factors, such as chronic diseases or obesity, which become more frequent with aging, can reduce HPG axis activity, disrupting the compensatory mechanisms and making hypogonadism clinically evident [3].

Work accompanies individuals of both genders for much of their lives and through all stages of adult life. The effects of the work environment combine with those of aging and of chronic conditions to determine the ultimate health status of the older person; occupational stress is an additive factor in the perception of menopause and LOH symptoms [4,5]. Menopause and LOH are both associated with increased cardiovascular mortality [6,7] and the loss of sex hormones; combined with age-associated immunosuppression and chronic inflammation, they may contribute to define the patterns of cancer incidence and mortality in the elderly [8]. 

In recent years, a longer life expectancy has been paralleled by a later retirement age, increasing the proportion of older people in the workforce. Since work, aging, and hormonal changes appear to be closely linked in the later stages of life, it is essential to understand the influence of work on menopause and LOH and the mechanisms underlying these phenomena. This review of the studies that have explored these aspects was conducted according to the PICO (Patient Intervention Comparison Outcome) model—P: general population or workers; I: aging and work-related conditions; C: comparison with no employment or between different types of employment; O: age at onset and signs/symptoms of andropause or menopause. We also assessed the quality of the available evidence according to the Cambridge Quality Checklists (CQCs).

## 2. Materials and Methods

This systematic review was performed according to the PRISMA statement [9].

### 2.1. Literature Search

The PubMed, SCOPUS and EMBASE databases were searched between January and February 2021. The search terms were “job”, “occupational”, “workers”, “workplace”, “occupational health”, “aging”, “hormones”, “testosterone”, “andropause”, and “menopause” in various combinations, as shown in Table 1. The references of the articles included in the first evaluation were screened for any additional relevant papers.

### 2.2. Inclusion Criteria 

Papers were considered eligible if they simultaneously addressed sex hormone levels or menopause, aging, and occupational status.

### 2.3. Exclusion Criteria

- Animal studies.
- Articles not in English.
- Reviews/conference abstracts or letters to the editor.
- Studies published before 2000 were excluded due to the vast changes—in terms of technology, tools, organization and methods and working environment—that have affected work over the past 20 years.

### 2.4. Studies Selection 

Two reviewers (M.M. and G.S.) independently evaluated the title, abstract and full text of each potentially relevant paper for eligibility and verbally resolved any discrepancies. A third reviewer (M.B.) was involved in the case of disagreement. The data extracted from the studies thus selected included the following: authors, year of publication, study design, and type, number, age, country, and hormone levels of the workers studied.

## 3. Results

**Figure 1:**
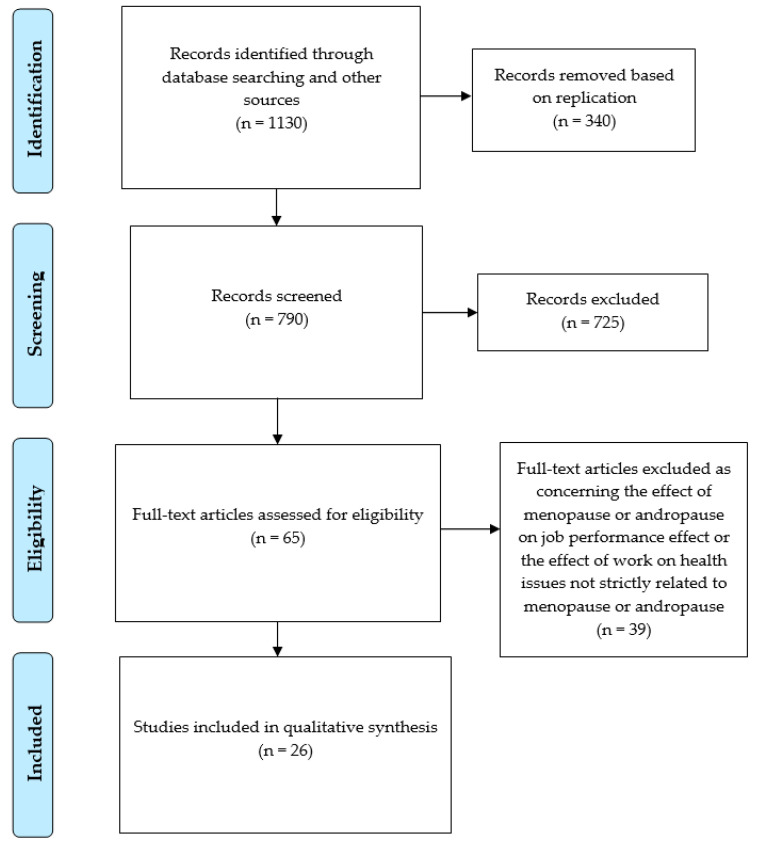
PRISMA flow diagram of the literature search.

All eligible studies examined the relationship among work, aging, and gonadal status (for men, both hormone levels and hypogonadism symptoms) and occupational status. The search strategy matched 1130 papers, of which 790 were evaluated after duplicates were removed. The title and/or abstract led to the exclusion of 725 articles. Of the remaining 65 papers, 26 matched the inclusion and exclusion criteria: 9 evaluated the effect of aging and work on male workers, 8 evaluated the effect of work on age at menopause onset, and 9 evaluated the effect of work on menopausal symptoms (Figure 1).

The results, sorted by gender, are reported below.

### 3.1. Aging and Gonadal Status in Male Workers

In a study of 664 blue-collar workers aged 40–60 years, Kratzik et al. explored the risk factors for hypogonadism by assessing a hormone panel in relation to age, body mass index (BMI), and the Aging Males’ Symptoms (AMS) questionnaire. The results were presented by AMS domain. For the psychological domain, the risk of a moderate-to-severe score significantly decreased with higher bioavailable T or total T (TT) levels; a similar result was described for the somatovegetative domain, where each year of age also significantly augmented (*p* < 0.001) the risk of a severe score; finally, age was the only significant predictor for the sexual domain [10]. 

Fukai et al. studied middle-aged Japanese office workers who had undergone an annual health check in 2002 and 2007. Their data were subjected to cross-sectional (96 and 76 men, respectively) as well as longitudinal analyses (33 men). Age was negatively associated with free T (FT; r = −0.399, *p* < 0.001 in 2002; r = −0.458, *p* < 0.001 in 2007) and dehydroepiandrosterone sulfate (DHEAS) (r = −0.233, *p* = 0.002 in 2002 and r = −0.336, *p* < 0.01 in 2007) levels, but not with TT. Moreover, in the longitudinal analysis, the 5-year changes in androgen levels were not significant [11].

In 2012, Hirokawa et al. investigated whether job demands modified the relationship between biochemical hypogonadism and andropause symptoms. To do so, they assessed the TT levels and health status in 183 middle-aged men (51.9 ± 7.7 years) working in a medium-sized company, who provided information about job demands and andropause symptoms, using the self-administered Job Content Questionnaire (JCQ) and the AMS scale. Subjects with T deficiency (TT < 12 nmol/L) showed a higher total score for andropause symptoms (*p* = 0.03) and psychological symptoms (*p* = 0.01). Moreover, age positively correlated with the sexual symptom and total andropause symptom scores, whereas job demands positively correlated with the AMS scores for total andropause symptoms, psychological symptoms, somatic symptoms, and sexual symptoms. Multiple regression analysis of andropause symptoms, age, and job demands showed a positive association with the total andropause score, whereas for job demands, the association remained significant also with somatic, psychological, and sexual symptoms. In a further stratified analysis based on job demands, men with high job demands showed an association of age and TT levels with the total score of andropause symptoms and with the somatic and psychological symptom scores [12]. 

Bann et al. conducted an extensive longitudinal study of a British cohort of 1780 subjects (875 men), who were examined 24 times from their birth in March 1946 to the age of 60–64 years, to assess whether socioeconomic position (SEP) in life could affect the hormone profile across multiple axes. According to one hormone measure from each axis, the authors found that among men aged 60–64 years, lower education and lower income were associated with lower FT levels, lower education was associated with lower insulin-like growth factor 1 (IGF-1) concentrations, and a lower lifetime SEP score was associated with higher evening cortisol. The fact that SEP exerted a stronger (and significant) effect on T levels at 60–64 years than at 53 years suggested to the authors that the socioeconomic patterning of endocrine function determinants may become especially pronounced in older subjects [13].

In 2016, Hirokawa et al. extended their previous evaluation to establish whether in 104 employees of a Japanese medium-sized company (2-year follow-up) changes in TT levels and/or job demands (as assessed by the JCQ) were associated with changes in andropause symptoms (as assessed by the AMS scale). They found that the changes in TT levels negatively correlated with changes in psychological symptoms, sexual symptoms, and the total AMS score, whereas changes in job demands positively correlated only with somatic changes. In a multiple regression model, the interaction of changes in job demands and TT levels was significantly associated with changes in psychological symptoms (standardized β = 0.26, *p* = 0.011). Age did not significantly affect either the changes in TT levels or the changes in AMS scores. The authors concluded that increased job demands may have moderating effects on the association between changes in TT levels and psychological symptoms [14].

A study by Łopuszańska-Dawid et al. included 300 healthy men aged 30–65 years belonging to three occupational categories: professionals (e.g., scientists, educators, physicians, lawyers, and managers), soldiers (e.g., sailors, customs officers, and armed bodyguards), and skilled workers (e.g., plumbers, mechanics, and hairdressers). The authors examined 17 measures of biological condition, including six hormones, and found that professionals had the highest values of IGF-1, calculated FT (cFT), and DHEAS, whereas skilled workers had the lowest TT, estradiol (E2), and DHEAS levels. A comparison of biological age and biological parameters showed that professionals and soldiers had a lower biological age than skilled workers. In all groups, chronological age correlated negatively and significantly with IGF-1, FT, E2, and DHEAS [15].

Pastuszak et al. enrolled 494 standard shift workers and 182 non-standard shift workers (whose shift began before 7:00 a.m. or after 2:00 p.m., rotated, or regularly included hours outside the standard 7:00 a.m.-to-6:00 p.m. workday) to assess the risk of hypogonadism symptoms and sexual dysfunction in relation to satisfaction with sleep quality (“very satisfied”, “somewhat satisfied”, “somewhat dissatisfied”, and “very dissatisfied”). Standard shift workers were generally significantly older than non-standard shift workers (*p* < 0.0001), whereas the Androgen Deficiency in the Aging Male (ADAM), quantitative Androgen Deficiency in Aging Male (qADAM), and international index of erectile function (IIEF) scores were not significantly different between the groups. Men working non-standard shifts and enjoying better sleep quality showed fewer hypogonadism symptoms and better sexual function, whereas sleep quality did not affect the hormonal profile. Hormones were also comparable in standard and non-standard shift workers [16].

The association between hypogonadism symptoms and sex hormone levels in relation to anthropometric and socioeconomic parameters was explored by Samipoor et al. in 140 men aged more than 40 years. The total AMS score was significantly related to occupation (*p* = 0.005), with more severe symptoms in self-employed subjects, compared to employees and workers. A similar relationship was observed for the somatic and psychological (but not the sexual) symptoms. The levels of LH and follicle stimulating hormone (FSH) increased with age, whereas there was no association between TT and FT levels and andropause symptoms [17].

Balasubramanian et al. examined the association between sleep disorders and hypogonadism symptoms in 619 non-standard shift workers (who began work before 7:00 a.m. or after 2:00 p.m., worked regularly outside the 7:00 a.m.-to-6:00 p.m. workday, or regularly rotated between standard and non-standard shifts) and 1952 daytime shift workers. A multivariate regression model (corrected for age, burden of comorbidities, T supplement use in the 2 weeks prior to the survey, and TT levels) indicated that non-standard shift workers at high risk of sleep disorders (*n* = 196) had significantly worse (*p* < 0.01) hypogonadism symptoms than non-standard shift workers without sleep disorders (*n* = 423) and that non-standard shift workers (regardless of the presence of sleep disorders) had significantly worse (*p* < 0.01) hypogonadism symptoms than daytime workers. In non-standard shift workers, sleep disorders were independently associated with lower TT levels (mean decrease, 100.4 ng/dL; *p* < 0.01) when controlling for age, comorbidities, and prior T supplementation. Moreover, age was a significant independent predictor of T level in shift workers with or without sleep disorders [18].

These data are summarized in Table 2. 

### 3.2. Aging and Gonadal Status in Female Workers

Whereas aging men who experience hypogonadism are a minority, all women exhaust ovarian function over time. The diagnosis of menopause can be confirmed by reduced blood estradiol, although it is more commonly based on at least 12 months of amenorrhea. Work influences menopausal status in two ways: age at onset and symptoms.

#### 3.2.1. Influence of Work on Menopause Onset

Physiological menopause occurs at an average age of 46 years [1], but some work-associated conditions can significantly modify age at onset.

According to Zhao et al., professional city women experience menopause at a later age (mean age, 48.2 ± 4.1 years) than women farmers living in rural areas (mean age, 46.5 ± 4.9 years). The effect of work on menopausal symptoms is also considered in their article [19] and is discussed below.

Based on experimental evidence of the ovotoxicity of some hormone-active pesticides, Farr et al. examined 8038 premenopausal women living or working on a farm and compared the age at menopause onset in those who had mixed or applied any pesticide (62%) versus unexposed women. Surprisingly, exposure to any pesticide was associated with a later age at menopause (hazard ratio (HR), 0.87), with an average increase of approximately 3 months in the median time to menopause, whereas exposure to hormonally active pesticides alone further increased this time by approximately 5 months [20].

The French ESTEV study, a prospective longitudinal epidemiological investigation that took place in 1990 and 1995, involved 1594 gainfully employed French women born in 1938, who experienced menopause at a median age of 52 years. In a recent analysis of these data, after distinguishing for a self-reported history of depression, the authors found that earlier age at menopause was associated with high job control (*p* = 0.03) and high school education (*p* < 0.01) in women with previous depression, whereas in those without previous depression, earlier age at menopause was associated with a high-strain job (*p* = 0.01) and difficult schedules (*p* = 0.03). Interestingly, in women without previous depression, later age at menopause was associated with repetitive work (*p* = 0.05), whereas depression itself was more frequent among women who reported exposure to high job strain (*p* < 0.04) and repetitive work (*p* < 0.004). Smoking (>10 cigarettes/day) was associated with an increased risk of earlier menopause in the entire sample (*p* < 0.001) [21].

A large cross-sectional survey conducted across Poland investigated 7183 women from all social strata to assess the correlation between menopausal age and demographic, social and lifestyle behavior. The median age at natural menopause was 51.25 years. Univariate Cox models were fitted to the age at menopause for all individual covariates before the multivariate analysis. Since employment was not significantly associated with age at menopause (HR, 0.92; 95% confidence interval [CI], 0.38–2.21), it was not included in the multiple Cox model. Cigarette smoking, low level of education, and a negative health perception were the only lifestyle behaviors that significantly correlated with earlier age at menopause [22].

In the NHANES III population, tobacco use (both primary and secondhand smoking) was associated with earlier age at menopause. Moreover, analysis of the employment status indicated that, although the difference was not statistically significant, service workers experienced the earliest age at menopause (46.95 years) and white-collar workers the latest (48.75 years). The authors suggested that secondhand smoking (which was frequent for service jobs) could play a role in earlier age at menopause onset [23]. Wang et al. also found an earlier age at menopause in blue-collar (49.0 years) than in white-collar workers (49.5 years), which they attributed to sulfur dioxide (SO_2_) exposure since occupational exposure to SO_2_ for more than 20 years was the only factor that remained significant on multivariate analysis [24].

The association between rotating night shift work and menopausal age was assessed by Stock et al. in a cohort of 80,840 nurses, with a follow-up of 22 years (1991–2013). During follow-up, 27,456 women (34%) experienced natural menopause at a mean age of 50 ± 4.0 years; those who in the previous 2 years had worked 20 or more months in rotating shifts had an increased risk of earlier age at menopause (HR, 1.09, 95% CI, 1.02–1.16), compared to non-shift working nurses. Cumulative rotating nightwork also exerted an effect since the younger nurses (<45 years) were at increased risk of earlier menopause (11–20 years: HR, 1.22, 95% CI, 1.03–1.44; ≥20 years: HR, 1.73, 95% CI, 0.90–3.35) than the older nurses [25].

In a cross-sectional community-based study of 425 women with a high rate of farming jobs (85.2%), Samtani et al. found that pesticide exposure induced a later, albeit not statistically significant, age at menopause [26].

These data are all summarized in Table 3.

#### 3.2.2. Influence of Work on Menopausal Symptoms

Several authors have examined the influence of work on the perception of menopausal symptoms.

In the study by Zhao et al., the prevalence of vasomotor symptoms was 35.1%; the prevalence of psychological symptoms was 78.5%; and the prevalence of bone/joint pain was 45.8%. After excluding premenopausal women, the prevalence of all symptoms was lower among postmenopausal women with farming jobs than among postmenopausal professional women; the prevalence of vasomotor symptoms was even lower among perimenopausal women with farming jobs [19].

A cross-sectional survey by Olaolorun et al. found a high prevalence of any menopausal symptom (84.5%); joint and muscular discomfort was the most common symptom (59.0%), followed by physical and mental exhaustion (43.0%), sexual problems (40.4%), and hot flashes (39.0%). Symptom perception was quantified using a structured questionnaire that included a standardized Menopause Rating Scale (MRS). Occupational status was defined as managerial and professional, intermediate, or routine and material. On multiple linear regression, occupation significantly predicted the total MRS score (HR, 0.76, 95% CI, 0.10–1.42) and the psychological subscale score (HR, 0.45, 95% CI, 0.16–0.75) [27].

Menopausal voice changes were investigated by Sovani et al. in 92 women divided into professional (teachers) and non-professional (clerks) voice users. The study analyzed various objective acoustic and aerodynamic parameters (fundamental frequency, relative average perturbation, shimmer, noise-to-harmonic ratio, voice turbulence index, speaking fundamental frequency and maximum phonation time) and subjective perception of voice changes (Voice Handicap Index). All subjects experienced menopausal voice changes irrespective of their job, although change severity and rate were greater in professional users, who were also aware of voice problems [28].

In 2010, Lee et al. investigated the factors associated with menopausal symptoms in a sample of 657 Korean women aged 41–59 years. On multivariable analysis, occupational status (“yes”/“no”) was significantly associated with the psychological subscale (β = −0.065, *p* < 0.05) and the total score (β = −0.058, *p* < 0.05) of the MRS [29].

In a survey by Oǧurlu et al., data from 132 women with a mean age of 48.9 ± 2.0 years were collected through the Climateric Complaint Tool, a questionnaire exploring 15 symptoms related to menopause. Based on occupational status, non-working women were more likely to report hot flashes, sleeping difficulties, headache, irritability, depressive mood, muscle and joint pain, and urinary complaints than were working women. Working status was also an independent predictor of the severity of climacteric symptoms on multivariate logistic regression analysis [30].

Hammam et al. investigated the relationship between work and menopausal symptoms in 131 middle-aged medical teaching staff workers. They found that several workplace factors, such as poor physical environment (91.6%), confined spaces/crowding (84.7%), insufficient sanitary/rest/refreshment facilities (83.2%), and poor workstation design (63.4%), worsened symptoms [31].

In Japan, Matsuzaki et al. examined 1169 female nurses aged 45–60 years (514 with and 655 without managerial positions) with two self-administered tools: Green’s Climateric Scale and the Brief Job Stress Questionnaire. They reported that high levels of job-related stress were associated with high scores on Green’s Climateric Scale, particularly for psychological symptoms; in particular, menopausal symptoms, such as general fatigue, irritability, and loss of concentration, were highly prevalent among nurses without managerial positions, whereas those with managerial positions reported more often feelings of unhappiness or depression due to psychological stress than the other nurses. In turn, nurses without managerial positions were more likely to report physical overload as a job-related stress factor. Having fewer interpersonal relationships significantly correlated with menopausal symptoms. Since the associations between job-related stress and vasomotor and somatic symptoms were weak (though significant) for all nurses, the authors suggested that minimizing job-related stress might reduce menopausal symptoms [32].

The effects of employment status, occupation, and education were explored by Huseth-Zosel et al. in a sample extracted from a longitudinal study, conducted to track the progression of metabolic syndrome in a Chinese urban population from 2008 to 2016. The authors reported that non-working women were less likely to have hot flashes, heart palpitations, and dry skin or eyes than women in jobs, whereas women with white-collar jobs were less likely to experience hot flashes, heart palpitations, or insomnia and were more likely to have dry skin or dry eyes than blue-collar workers and non-working women, although none of these differences were statistically significant. Moreover, women in jobs were more likely to report musculoskeletal pain and less likely to report neurological symptoms or emotional distress than non-working women, whereas the latter women were more likely than women with white-collar and blue-collar jobs to experience emotional distress, musculoskeletal pain, and neurological symptoms (*p* < 0.05) [33].

Bariola et al. recently tested the association of menopausal symptom severity and employment conditions (e.g., employment status, flexible working hours, temperature control, job autonomy, and supervisor support) in an online survey of 476 peri- and postmenopausal women (mean age, 53.69 years) working in higher education in Australia. According to multivariable linear regression, reporting of lower menopausal symptom was associated with greater supervisor support (β = −0.10, *p* = 0.04), full-time job (β = −0.11, *p* = 0.02), and control over temperature (β = −0.11, *p* = 0.02), whereas no association emerged with job autonomy or flexible working hours [5].

These data are summarized in Table 4.

#### 3.2.3. Quality of the Evidence

To evaluate the quality of the available evidence, the studies included in the systematic review were subjected to the CQCs as described by Murray et al. [34]. The CQCs consist of three scales with different scores: score correlate (0–5), risk factor score (1–3), and causal risk factor score (1–7). The results are reported in Table 5.

With regard to the studies investigating work and andropause, the overall quality of correlates was poor, partly due to the small sample size and to inadequate sampling methods. The majority of studies had a cross-sectional design, whereas three (33.3%) had a prospective design. None included a control group.

In the studies exploring work and age at menopause, the correlate score was slightly better because the works involved larger populations; moreover, four (50%) had a cross-sectional design, one (12.5%) had a retrospective design and three (37.5%) had a prospective design.

Finally, the correlate score of studies investigating work and menopausal symptoms was intermediate. All these studies had a cross-sectional design.

## 4. Discussion

There is little information on the correlation between work and aging-related hormonal alterations. This systematic review of papers published from 2000 to 2020 identified 26 studies assessing sex hormone levels (andropause or menopause) in relation to occupational status. The overall quality of the published data is poor, and most of the information comes from uncontrolled, prospective or cross-sectional studies. In addition, most of the samples are from specific groups of workers, and participant selection is not randomized, which clearly involves a high risk of selection bias. The main tools used in the studies were questionnaires and direct comparison between hormonal status and occupational factors.

The investigation of the relationship between work-related factors and andropause/menopause was not the focus of all 26 studies since some analyzed very specific occupational factors, whereas others asked only generic questions about the type of work.

All studies involving men measured T or TT levels in relation to age, whereas some also analyzed DHEAS [11,15], IGF-1 [13,15], BMI [10], type of work [12,14,15,16,17,18], andropause symptoms (with the AMS scale [12,14]), job content (with the JCQ questionnaire [12,14]), lifetime SEP [13], and sleep disorders [16,17].

There was a positive correlation between age and the severity of andropause symptoms and psychological symptoms [10,12]. Two investigations found that age negatively correlated with FT and DHEA [11,15] but not with TT [11], whereas another did not confirm the correlation between age and FT level [14].

Based on the type of work, professionals showed higher levels of IGF-1, FT, and DHEAS than skilled workers and soldiers [15], while skilled workers showed lower TT and DHEAS levels, compared to the other job categories. Job demands and lifetime SEP also seem to affect TT levels and andropause symptom severity [12,13,14]. Sleep quality was investigated in standard and non-standard shift workers [16,18] and was associated with TT levels in one study [16], even though both works reported a positive correlation between sleep disorders and andropause symptom severity.

The variables investigated in the studies exploring the influence of work on age at menopause onset were smoking [21,22,23,24], type of work [19,21,22,23,24], shiftwork [25], and use of pesticides [20,26].

A positive correlation between smoking habits and earlier age at menopause onset was found in all studies [21,22,23,24]. Menopause onset was earlier in blue-collar than in white-collar workers [19,24], suggesting a possible incidence of prolonged physical effort on menopausal age. High job strain, repetitive work, and the use of pesticides seem to lead to an earlier age at menopause onset [20,21,26]. Finally, one work described an association between rotating shiftwork and earlier menopause [25]. In addition, the occupational status was significantly associated with menopausal symptoms in all studies [27,28,29,30,33]. Notably, high levels of work-related stress were associated with psychological symptoms, while physical overload was more likely to be associated with manual work [32]. No association was reported between flexible working hours and menopausal symptoms [5].

The review shows the possible influence of work on TT, FT, IGF-1, and DHEA alterations and on andropause symptom severity in men; in particular, some studies described a correlation, with work-related stress load on the one hand and with physical effort on the other.

In women, a correlation was described among high job strain, repetitive work and anticipation of menopause onset and menopausal symptom severity. This should be taken into consideration, especially by occupational physicians in their health surveillance activity. Age-based organization of the workload or health promotion programs would be desirable.

Sleep disorders seem to affect both hormone levels and andropause symptom manifestations, suggesting that, as in women, shift work can modify circadian rhythms and affect hormone synthesis. Since these alterations have the potential to lead to age-related disease onset, sleep disorders should be investigated in worker health surveillance, especially in shift workers. More in-depth studies are needed.

Work accompanies men and women throughout most of their lives. Despite this, the quality of the available evidence is scarce. Moreover, the vast majority of the data come from cross-sectional studies, whereas correct assessment of the role of a variable (such as work) as a risk factor requires longitudinal studies. The monitoring of modifiable factors, related to the workplace or the organization of work, that can affect worker health also has the potential to reduce absenteeism and enhance well-being in the workplace and productivity.

## 5. Conclusions

The available information on the correlation between work and aging-related hormonal alterations is limited. The studies conducted to date do not rule out an influence of work on andropause or menopause. Some occupational factors, such as job-related stress, sleep disorders related to shift work, and pesticide exposure, are implicated in aging-related hormonal alterations. The possible role of work on andropause/menopause warrants further investigation and greater attention by the scientific community.

## Figures and Tables

**Table 1:** Search strategy adopted to identify relevant articles.

1—“aging” AND “workers” AND “hormones”
2—aging” AND “workers” AND “testosterone”
3—“aging” AND “workers” AND “andropause”
4—“aging” AND “workers” AND “menopause”
5—“aging” AND “occupational” AND “hormones”
6—“aging” AND “occupational” AND “testosterone”
7—“aging” AND “occupational” AND “andropause”
8—“aging” AND “occupational” AND “menopause”
9—“aging” AND “job” AND “hormones”
10—“aging” AND “job” AND “testosterone”
11—“aging” AND “job” AND “andropause”
12—“aging” AND “job” AND “menopause”
13—“andropause” AND “occupational” AND “workers”
14—“menopause” AND “occupational” AND “workers”
15—“workplace” AND “aging” AND “hormones”
16—“workplace” AND “aging” AND “testosterone”
17—“workplace” AND “aging” AND “andropause”
18—“workplace” AND “aging” AND “menopause”
19—“occupational health” AND “aging” AND “hormones”
20—“occupational health” AND “aging” AND “testosterone”
21—“occupational health” AND “aging” AND “andropause”
22—“occupational health” AND “aging” AND “menopause”
23—“andropause” AND “job”
24—“menopause” AND “job”

**Table 2:** Studies evaluating work and andropause.

Reference	Study Design	Worker Type	No. of Subjects	Age	Country	Hormones Evaluated	Aim of the Study	Outcome Measures	Additional Tools	Results
Kratzik et al., 2004 [10]	Cross-sectional	Blue-collar workers	664	40–60	Austria	LH, T, E2, PRL, SHBG, BAT	To explore the relationship between hormonal status and the AMS score, taking into consideration the influence of BMI and age on hormonal status	AMS	BMI	Low T or BAT levels involved a significantly increased risk of psychological symptoms of andropause. Low T and BAT, together with aging, increased the risk of somatovegetative symptoms, whereas aging increased the risk of sexual symptoms.
Fukai et al., 2010 [11]	Cross-sectional * and longitudinal ** (FU 5 years)	Office workers	139 (96 and 76 *, 33 **)	52.7 ± 5.9 and 51.5 ± 6.8 * 54.3 ± 5.4 **	Japan	TT, FT DHEAS,	To assess age-related changes in plasma androgen levels in healthy middle-aged men and elucidate whether any clinical parameters measured in health check-ups were associated with hormonal changes	Blood pressure, BMI, waist circumference, lipid panel		Age was negatively associated with FT and DHES, but not with TT. In the longitudinal survey, the 5-year changes in androgen levels were not significant.
Hirokawa et al., 2012 [12]	Cross-sectional	Employees of a medium-sized company	183	51.9 ± 7.7 (34–67)	Japan	TT	To establish whether job demands modify the association between low T levels and andropause symptoms	Japanese version of JCQ, AMS, hormonal parameters	Information on history of lifestyle diseases, smoking status, alcohol consumption and sleeping hours	Low TT levels were associated with more psychological andropause symptoms. High job demands, age, and TT levels were positively associated with the total score of andropause symptoms and with somatic and psychological symptom scores.
Bann et al., 2015 [13]	Longitudinal (FU 60–64 years)	Professional, intermediate, skilled (non-manual), skilled (manual), partly skilled, unskilled	875	60–64	U.K.	IGF-1, IGF-2, IGFBP-3, TT, SHBG, cFT, evening salivary cortisol	To test the hypothesis that lower SEP across life could be associated with an adverse hormone profile across multiple axes	SEP indicators across life: paternal occupation at 4 yo, highest educational level at 26 yo, highest occupational class at 53 yo, self-reported household income at 60–64 yo		At 60–64 years, lower education and lower income were associated with lower T levels, lower education was associated with lower IGF-1, and a lower lifetime SEP score was associated with higher evening cortisol. The socioeconomic patterning of endocrine function determinants may become more pronounced in older men.
Hirokawa et al., 2016 [14]	Longitudinal (FU 2 years)	Employees of a medium-sized company	104	52.8 ± 7.2 (36–62)	Japan	TT	To establish whether changes in job demands modify associations between changes in T levels and andropause symptoms	Japanese version of JCQ, AMS, hormonal parameters	Information on past history of lifestyle diseases, smoking status, alcohol consumption and sleep hours	Changes in T levels correlated negatively with changes in psychological symptoms, sexual symptoms, and total AMS score, whereas changes in job demands positively correlated only with somatic changes. The interaction of changes in job demands and T levels was associated with changes in psychological symptoms.
Łopuszańska-Dawid et al., 2016 [15]	Cross-sectional	Professionals, soldiers, and skilled workers	300	30–65	Poland	IGF-1, TT, cFT, SHBG, E2, DHEAS	To investigate the relationship between occupational activity and biological condition in adult men	11 measures of general biological condition, 6 hormonal parameters	Data on socioeconomic status, lifestyle, type of work, general life satisfaction (low, medium, high), and approach to life (optimist, pessimist, mixed)	Relative biological age with reference to the general and hormonal parameters was lowest in professionals and highest in skilled workers. Professionals had the highest IFG-1, cFT, and DHEAS values, whereas skilled workers had the lowest TT, E2, and DHEAS values
Pastuszak et al., 2017 [16]	Cross-sectional	Shift workers	676 (182 non-standard shift workers ° and 494 standard shift workers °°)	41.4 ± 10.8 ° 46.4 ± 14.7 °°	U.S.A.	TT, FT, E2, DHEA, FSH, LH	To evaluate the impact of sleep quality on hypogonadism symptoms and sexual function in standard and non-standard male shift workers	ADAM, qADAM, IIEF, self-reported satisfaction with sleep quality		In non-standard shift workers, better sleep quality was associated with fewer hypogonadism symptoms and a better sexual function. Sleep quality did not affect hormone levels, which were comparable in standard and non-standard shift workers.
Samipoor et al., 2018 [17]	Cross-sectional	Self-employed subjects, employees, and workers	140	52.1 ± 7.1	Iran	TT, FT, FSH, LH	To clarify the association between hypogonadism symptoms and sex hormone levels, according to anthropometric and socioeconomic parameters (including occupation)	AMS	Information on history of lifestyle diseases, smoking status, alcohol consumption and blood pressure	No significant association between T levels and andropause symptoms. Significant association between occupation and hypogonadism symptoms, with the highest score in self-employed subjects
Balasubramanian et al., 2020 [18]	Cross-sectional	Shift workers versus daytime workers	2571	42.9 (weighted average)	U.S.A.	TT, FSH, LH	To examine the association among shift work, sleep disorders and hypogonadism symptoms in shift workers	qADAM, ADAM	RAPA, CCI, PHQ-9	Non-standard shift workers showed worse hypogonadism symptoms than daytime workers; the further presence of sleep disorders was associated with even worse hypogonadism symptoms and lower T levels. Age was a significant independent predictor of T level in shift workers with or without sleep disorders.

* Cross-sectional; ** Longitudinal; ° Non-standard shift workers; °° Standard shift workers ADAM = Androgen Deficiency in the Aging Male; AMS = Aging Males’ Symptoms; BAT = bioavailable testosterone; BMI = body mass index; CCI = Charlson Comorbidity Index; cFT = calculated free testosterone; DHEA = dehydroepiandrosterone; DHEAS = dehydroepiandrosterone sulfate; E2 = estradiol; FSH = follicle stimulating hormone; FT = free testosterone; FU = follow-up; IIEF = International Index of Erectile Function; IGF-1 = insulin-like growth factor 1; IGF-2 = insulin-like growth factor 2; IGFBP-3 = insulin-like growth factor binding protein 3; IIEF = international index of erectile function; yo = years old; JCQ = Job Content Questionnaire; LH = luteinizing hormone; PHQ-9 = Patient Health Questionnaire-9; PRL = prolactin; qADAM = quantitative Androgen Deficiency in the Aging Male; RAPA = Rapid Assessment of Physical Activity; SEP = socioeconomic position; SHBG = sex hormone-binding globulin; T = testosterone; TT = total testosterone.

**Table 3:** Studies evaluating work and age at menopause.

Reference	Study Design	Worker Type	No. of Subjects	Country	Exposure	Results
Zhao et al., 2000 [19]	Cross-sectional	Professionals and farmers	806	China	Rural environment	Professional city women experienced a later age at menopause than women farmers living in rural areas.
Farr et al., 2006 [20]	Longitudinal prospective	Farm workers	8038	U.S.A.	Pesticides	The median time to menopause increased by 3 months for any pesticide exposure and by 5 months for hormonally active pesticides.
Cassou et al., 2007 [21]	Longitudinal prospective	Gainfully employed women	1594	France	Occupational factors	Occupational factors, such as job control or high-strain jobs and difficult schedules, were associated with earlier age at menopause in women with and without a history of depression, respectively. Smoking increased the risk of earlier menopause.
Kaczmarek et al., 2007 [22]	Cross-sectional	Not specified	7183	Poland	Occupation	Occupational status is not a risk factor for earlier age at menopause.
Fleming et al., 2008 [23]	Cross-sectional	White collar, service, farm workers, blue collar	5029	U.S.A.	Smoking	Primary tobacco use and secondhand smoke were associated with earlier menopause. Service and manufacturing industry workers had a greater potential secondhand smoke exposure and showed earlier age at menopause (not significant).
Wang et al., 2015 [24]	Retrospective cohort	White collar, blue collar	3167	China	Sulfur dioxide	Blue-collar workers experienced earlier menopause than white-collar workers. Exposure to sulfur dioxide (especially for 21 to 25 years) was associated with earlier age at menopause.
Stock et al., 2019 [25]	Longitudinal prospective	Rotating night shift nurses	80,840	U.S.A.	Night shifts	Rotating night shift work was associated with an increased risk of earlier menopause, especially among women aged < 45 years.
Samtani et al., 2020 [26]	Cross-sectional	Agriculture, other	425	India	Pesticides	Pesticide exposure was associated with older age at menopause (not significant).

**Table 4:** Studies evaluating work and menopausal symptoms.

Reference	Study Design	Worker Type	No. of Subjects	Country	Symptoms	Results
Zhao et al., 2000 [19]	Cross-sectional	Professional workers and farmers	806	China	Vasomotor and psychological symptoms, bone/joint pain	In the peri- and postmenopausal period, professional city women showed a higher prevalence of menopausal symptoms than women farmers living in rural areas.
Olaolorun et al., 2009 [27]	Cross-sectional	Managerial and professional, intermediate, or routine and material workers	1189	Nigeria	Joint and muscular discomfort, physical and mental exhaustion, sexual problems, hot flashes	Occupation significantly correlated with menopausal symptoms, especially with psychological symptoms.
Sovani et al., 2010 [28]	Cross-sectional	Professional (teachers) and non-professional (clerks) voice users	92	India	Voice changes	All women exhibited menopausal voice changes, but professional voice users presented a higher severity and rate of changes.
Lee et al., 2010 [29]	Cross-sectional	Non-employed, employed	657	Korea	Menopause Rating Scale	Employed women with jobs had less severe menopausal symptoms than non-employed women
Oǧurlu et al., 2011 [30]	Cross-sectional	Non-employed, employed	132	Turkey	Climateric Complaint Tool (15 symptoms)	Non-working women were more likely to report hot flashes, sleeping difficulties, headache, irritability, depressive mood, muscle and joint pain, and urinary complaints than working women. Working status was an independent predictor of the severity of climacteric symptoms on multivariate logistic regression analysis.
Hammam et al., 2012 [31]	Cross-sectional	Medical teaching staff	131	Egypt	Depressed mood, vasomotor symptoms, anxiety, sexual and sleep problems	Several workplace factors (poor physical environment, confined spaces/crowding, insufficient sanitary/rest/refreshment facilities, and poor workstation design) worsened menopausal symptoms.
Matsuzaki et al., 2014 [32]	Cross-sectional	Nurses	1169	Japan	Green’s Climateric Scale (21 symptoms)	High levels of job-related stress significantly correlated with menopausal symptoms in all nurses, although stressors were different for those with and without managerial positions.
Huseth-Zosel et al., 2014 [33]	Cross-sectional	Non-employed, white-collar and blue-collar workers	296	China	Hot flashes, dry skin/eyes, sleeplessness, heart palpitations, musculoskeletal pain, neurological symptoms, emotional distress	Employment, particularly a white-collar job, exerted some protective effect on certain menopausal symptoms.
Bariola et al., 2017 [5]	Cross-sectional	Higher education workers	476	Australia	Menopause Symptom Index (19 symptoms)	Lower menopausal symptom reporting was associated with workplace conditions such as greater supervisor support, working full, and control over temperature.

**Table 5:** Cambridge Quality Checklist scores of the studies included in the systematic review.

	Work and Andropause (n = 9)	Work and Age at Menopause (n = 8)	Work and Menopausal Symptoms (n = 9)
**Correlate score**			
0	0	0	0
1	3	0	1
2	3	0	3
3	3	8	5
4	0	0	0
5	0	0	0
**Risk factor score**			
1. Cross-sectional data	6	4	9
2. Retrospective data	0	1	0
3. Prospective data	3	3	0
**Causal risk factor score**			
1. Study without a comparison group No analysis of change 2. Inadequately controlled study No analysis of change 3. Study without a comparison group With analysis of change 4. Inadequately controlled study With analysis of change 5. Controlled non-experimental study No analysis of change 6. Controlled non-experimental study With analysis of change 7. Randomized experiment Targeting a risk factor	6 0 3 0 0 0 0	8 0 0 0 0 0 0	9 0 0 0 0 0 0
